# Massachusetts Medical College

**Published:** 1850-11

**Authors:** 


*Massachusetts Medical College.*—Dr. E. N. Horsford has been appointed Professor in Chemistry in this Institution.

